# Dataset on early-age strength of ambient-cured geopolymer mortars from waste concrete and bricks with different alkaline activators

**DOI:** 10.1016/j.dib.2024.110800

**Published:** 2024-08-06

**Authors:** Reginald B. Kogbara, Abdelrahman Al-Zubi, Eyad A. Masad

**Affiliations:** a Mechanical Engineering Program, Texas A&M University at Qatar, P.O. Box 23874, Education City, Doha, Qatar; b School of Engineering, University of Greenwich, Medway Campus, Chatham, ME4 4TB, UK; c Chemical Engineering Program, Texas A&M University at Qatar, P.O. Box 23874, Education City, Doha, Qatar; d Zachry Department of Civil & Environmental Engineering, Texas A&M University, College Station, TX 77843, USA

**Keywords:** Alkali-activated mortar, Brick waste, Compressive strength, Concrete waste, Early-age strength, One-part geopolymer, Solid-powder alkaline activator

## Abstract

The dataset presented here emanates from preliminary studies that compared the early-age compressive strengths of geopolymer mortars produced from construction and demolition wastes (CDW) commonly found in Qatar using different alkaline activators. Waste concrete, waste bricks and steel slag were used as aluminosilicate sources for the geopolymer mortars. Waste concrete was used as fine aggregate (75 µm to 4 mm), while solid or hollow red clay bricks were used together with steel slag as aluminosilicate powders. Solid red clay brick (75 µm to 1.4 mm) was also considered as fine aggregate. Different alkaline activators including solid powder or ground pellet forms of Ca(OH)_2_, CaO, and Ca(OH)_2_-NaOH, NaOH-CaCO_3_ and Na_2_SiO_3_-Na_2_CO_3_-Ca(OH)_2_ mixtures were employed by just adding water. Both solid powder Ca(OH)_2_ and viscous solutions of NaOH and NaOH-Na_2_SiO_3_ were also considered as alkaline activators. The geopolymer mortars included small amounts of some other additives such as gypsum, microsilica and aluminium sulfate to enhance the geopolymerization and hydration process. Random proportions of the materials were employed in the range-finding experiments, and the mortars produced were tested for compressive strength. The dataset shows the 7-day compressive strengths and densities of the 40 mixtures tested with mostly ambient temperature (20°C) curing. It also shows such data for mixtures in which variables such as curing at 40°C, mixing with hot water at 50 - 60°C temperature, grading of waste concrete aggregates, and collective grinding of the powdered materials were considered. The data indicates possible early-age compressive strengths of different geopolymer mortar mixture designs and the materials and mixture design methods that can be used to achieve desired early-age strengths from waste concrete and bricks.

Specifications Table

**Table:** 

Subject	Civil and Structural Engineering
Specific subject area	*Construction materials*
Type of data	Table (.xlsx format), Image (.jpg format), Figure (.xlsx format), Analyzed; Software used: Microsoft Excel Version 2406(Build 17726.20160)
Data collection	Data were collected through laboratory measurements, standard test sieves, 2.25×3 inches model LC-27F-O363835 laboratory jaw crusher, 50×50×50 mm molds, Hobart N50 5-Quart commercial stand mixer, Thermo Lindberg/Blue M oven, 450-W Philips model HR2058/91 blender*.*
Data source location	Doha, Qatar.
Data accessibility	The data is within this article, and the raw data leading to the processed data presented in Tables 1 – 3 and Figure 1 in MS Excel format is deposited on Figshare [1]: Repository name: Figshare Data identification number: 10.6084/m9.figshare.25998328 Direct URL to data: https://doi.org/10.6084/m9.figshare.25998328.v1 Data are available under the terms of the Creative Commons Zero ``No rights reserved'' data waiver (CC0 1.0 Public domain dedication).
Related research article	None

## 1 Value of the Data

• The data is valuable as it provides information on different mix design methodologies that can be used to develop geopolymers for use as construction materials from construction and demolition wastes (CDW) with predominantly concrete waste, which is not commonly used for geopolymers as it could have detrimental impact on compressive strength in contrast to mixtures with larger amounts of other CDW such as brick waste. Such mix design methodologies include the use of graded concrete aggregates, mixing with hot water at elevated temperatures (50°C, 55°C and 60°C) and collective grinding of the powdered materials.
• The dataset provides some basis to compare the early-age strength of one-part geopolymers (just adding water to a mixture of solid alkaline activator(s) and aluminosilicate powders) and two-part geopolymers (conventional with viscous alkaline activator solutions) as it includes some two-part geopolymer mixtures although most of the mortar mixtures are one-part geopolymers. This can help in making informed choices of the type of geopolymer (whether one- or two-part) to use for a given application - for example, 3D printing and retrofitting applications, including cladding and addition of aesthetic features.
• Since about 80 – 90% of the 28-day compressive strength of slag-based geopolymers is achieved within 7-days of curing, and compressive strength is a crucial parameter in concrete structure design, the dataset would be useful to researchers and practitioners in the field of construction materials dealing with geopolymer mortars. These include entities interested in mortars for 3D concrete printing, and stakeholders with green building goals such as owners, investors, developers, contractors, designers, engineers, consultants, etc. The dataset can help interested parties identify green geopolymer mortar mixtures from predominantly concrete waste to use or improve upon for a given application, especially in areas where concrete waste is the most abundant CDW.

## 2 Background

Construction and demolition wastes (CDW), especially waste concrete and bricks, generally contain aluminosilicates since their constituent materials contains alumina and silicates. Hence, they can be used as raw materials for geopolymer production. Compared to conventional cementitious materials, geopolymers have high early strength and good durability [2]. Consequently, an investigation was carried out to produce geopolymer mortars from CDW commonly available in Qatar (mainly waste concrete and small amounts of red clay bricks) and to utilize such mixtures for additive manufacturing (3D concrete printing – 3DCP) [3]. Waste concrete was the majority component in the geopolymer mortar mixtures since it is the most abundant CDW in Qatar [4]. Recent studies have shown the utility of recycled concrete aggregates in new concrete/geopolymer mixtures [[5], [6], [7]].

The data described emanates from preliminary range-finding experiments in which random proportions of component materials for the geopolymer mixtures were considered with a view to improving upon high early-age strength mixtures for use in 3DCP. It is documented that around 80 - 90% of the 28-day compressive strength of slag-based geopolymers can be achieved within 7 days of curing [8]. Hence, this preliminary work focused on early-age (7-day) compressive strengths of geopolymer mortars with different mix design methodologies.

## 3 Data Description

**Fig. 1:**
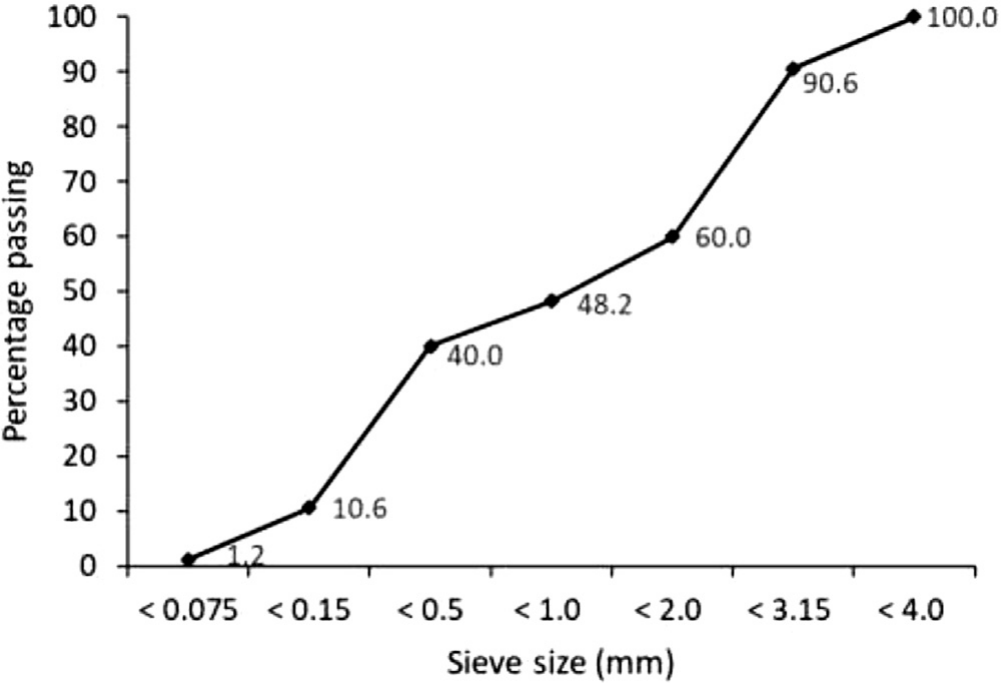
Sieve analysis curve of the waste concrete aggregates in mixes that employed graded waste concrete aggregates.

**Fig. 2:**
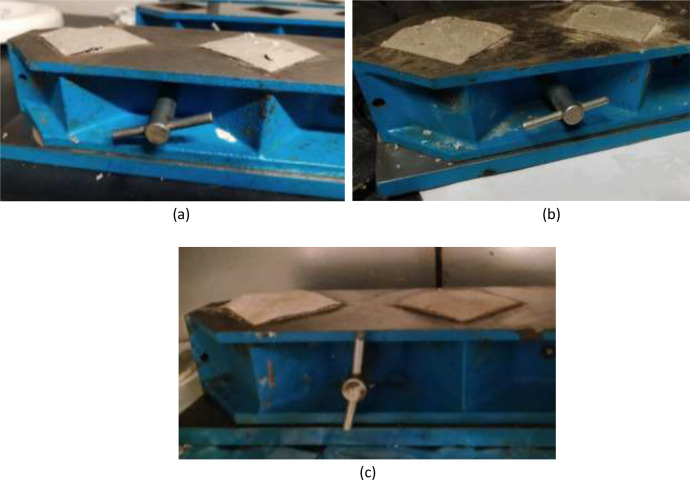
Mixes 19, 26 and 27 in (a), (b) and (c), respectively, with CaO as sole alkaline activator showing some form of swelling or heaving a few hours after casting.

The data presented in Table 1, Table 2, Table 3 show the 7-day compressive strengths and densities of the 40 mixtures considered, mostly with ambient temperature (20°C) curing. It also shows the same data for some mixtures with variables such as curing at 40 or 50°C (see Mixes 33, 35, 38 in Table 2, and Mixes 39 and 40 in Table 3), mixing with hot water at 50 - 60°C temperature (Mixes 14, 15, 19, 24, 26, 27 in Table 1), graded waste concrete aggregates (Mixes 7, 9 - 12, 17, 18 and 21 in Table 1), collective grinding of powdered materials (Mixes 29 – 38 in Table 2 and Mixes 39 and 40 in Table 3) and inclusion of waste glass powder (Mixes 36 and 40, Table 2, Table 3). Fig. 1 shows the sieve analysis curve of the graded waste concrete aggregates employed in applicable mixes. The mixes with CaO as sole alkaline activator generally showed some form of swelling or heaving a few hours after casting as shown in Fig. 2 and the top surface was removed and the mix re-casted, which is like a previous report of expansion of perlite geopolymer concrete specimens with nano-CaO inclusion [9]. The dataset in this work shows possible early-age compressive strengths of different geopolymer mortar mixture designs and the materials and mixture design methods that can be used to achieve desired early-age strengths from waste concrete and bricks*.*

**Table 1:** Mix design (percent by weight) and 7-day compressive strengths and densities of geopolymer mortars with solid powdered lime-based {Ca(OH)_2_ or CaO} single alkaline activators mostly cured at ambient temperature.

Mix Number	Aluminosilicate sources					Alkaline activators		Additives			Superplasticizer	Total	Specific mix design Comment	Water/Cementitious materials ratio	Average density (kg m^−3^)	Average compressive strength (MPa)
	Steel slag	Waste solid brick powder	Waste hollow brick powder	Waste concrete aggregate	Waste solid brick aggregate	Ca(OH)_2_ powder	CaO powder	Al_2_(SO_4_)_3_.18H_2_O	Gypsum	Microsilica						
1	16	–	–	71	–	7	–	2	–	3.8	0.2	100	–	0.47	1944	17
2	18	–	–	70	–	7.8	–	–	–	4	0.2	100	–	0.48	1976	13
3	18	–	–	70	–	–	7.8	–	–	4	0.2	100	–	0.45	1784	8
4	20.8	–	–	70	–	–	5	–	–	4	0.2	100	–	0.60	2016	15
5	20	–	–	70	–	–	5.8	–	–	4	0.2	100	–	0.60	2032	15
6	21.6	–	–	70	–	–	4.3	–	–	3.9	0.2	100	–	0.60	2080	14
7	17.2	–	–	68	–	7.5	–	3	0.5	3.6	0.2	100	Graded concrete aggregate	0.50	2112	12
8	17	–	–	68	–	7.5	–	3	0.5	3.8	0.2	100	–	0.70	1832	19
9	17	–	–	68	–	7.5	–	3	0.5	3.8	0.2	100	Graded concrete aggregate	0.65	1888	18
10	16.9	–	2.4	67	–	7	–	6.5	–	–	0.2	100	Graded concrete aggregate	0.52	1952	22
11	17	–	–	66.5	–	7.3	–	6	–	3	0.2	100	Graded concrete aggregate	0.53	1936	17
12	21	–	4.5	65	–	9.2	–	–	–	–	0.3	100	Graded concrete aggregate	0.50	2056	15
13	21	–	4.5	65	–	9.2	–	–	–	–	0.3	100	–	0.47	2064	20
14	21	–	4.5	65	–	9.2	–	–	–	–	0.3	100	Mixed using hot water at 50°C	0.47	2064	17.5
15	21	–	–	60	9.5	–	9.2	–	–	–	0.3	100	Mixed using hot water at 60°C	0.47	1920	16.5
16	28	5	–	50	–	14	–	–	0.6	2	0.4	100	–	0.43	1907	21
17	24.5	–	–	45	–	12.3	–	12	1.5	4.4	0.3	100	Graded concrete aggregate	0.54	1680	15
18	28	–	–	45	–	14	–	2.6	2.5	7.5	0.4	100	Graded concrete aggregate	0.56	1816	21
19	28.9	–	–	45	20	–	5.8	–	–	–	0.3	100	Mixed using hot water at 55°C	0.45	1984	11
20	29.6	10	–	45	–	14.9	–	–	–	–	0.5	100	–	0.43	1884	13
21	29.5	–	–	44.8	–	14	–	1.6	2.5	7.3	0.3	100	Graded concrete aggregate	0.54	1800	23.5
22	30	–	–	40	–	15	–	4.6	–	10	0.4	100		0.40	1744	17
23	26.5	–	–	40	–	13	–	4.5	5	10	1.0	100		0.41	1624	10.5
24	28.4	–	–	40	25	–	6.3	–	–	–	0.3	100	Mixed using hot water at 60°C	0.44	2056	16
25	20	–	10	35	–	20	–	7.5	–	7.5	–	100	–	0.47	1528	10
26	28.4	–	–	35	30	–	6.3	–	–	–	0.3	100	Mixed using hot water at 50°C	0.44	1960	15
27	28.4	–	–	35	30	–	6.3	–	–	–	0.3	100	Mixed with normal water, cured at 50°C for 24 hours	0.46	1877	12.5
28	0	–	48	14	–	25	–	9	4	–	–	100	–	0.46	1248	4.5

*Note:* Samples were cured at ambient temperature unless stated otherwise in the specific mix design comment.

**Table 2:** Mix design (percent by weight) and 7-day compressive strengths and densities of geopolymer mortars with solid powdered NaOH/Na_2_SiO_3_-based binary or ternary alkaline activators mostly cured at ambient temperature.

Mix Number	Aluminosilicate sources				Alkaline activators					Additives		Superplasticizer	Total	Specific mix design Comment	Water/Cementitious materials ratio	Average density (kg m^−3^)	Average compressive strength (MPa)
	Steel slag	Waste solid brick powder	Waste concrete aggregate	Waste solid brick aggregate	Ca(OH)_2_ powder	Ground NaOH pellets	CaCO_3_ powder	Na_2_SiO_3_ powder	Na_2_CO_3_ powder	Gypsum	Microsilica						
29	20.9	5	50	10	10.4	3.4	–	–	–	–	–	0.3	100	Equivalent NaOH molarity = 4.8	0.45	2090	23
30	18	5	50	10	12.1	4.9	–	–	–	–	–	–	100	Equivalent NaOH molarity = 6.8	0.45	2010	16
31	17.1	5	50	10	11.4	6.5	–	–	–	–	–	–	100	Equivalent NaOH molarity = 9.0	0.46	2102	14.5
32	17.4	5	50	10	10.8	6.8	–	–	–	–	–	–	100	Equivalent NaOH molarity = 6.7	0.64	1931	9
33	31	3	50	7	–	4	5	–	–	–	–	–	100	Equivalent NaOH molarity = 5.0, cured at 40°C for 7 days	0.48	1882	18
34	25	3	47	5	12.5	4	–	–	–	1.5	1.6	0.4	100	Equivalent NaOH molarity = 5.0	0.42	2038	21.5
35	–	20	47	10.1	12	10.9	–	–	–	–	–	–	100	Equivalent NaOH molarity = 16.8, cured at 40°C for 7 days	0.38	2037	9.5
36	5⁎⁎	3.3	43.5	18.6	8.2	–	–	14	7	–	–	0.4	100	–	0.37	1912	6
37	26.4	3	40	17	10.2	3	–	–	–	–	–	0.4	100	Equivalent NaOH molarity* = 4.1	0.43	2063	24
38	36.4	3	40	17	–	1.6	2	–	–	–	–	–	100	Equivalent NaOH molarity = 2.0, cured at 40°C for 7 days	0.47	1874	21

*Note:* There was collective grinding of the powdered materials for all mixes. Samples were cured at ambient temperature unless stated otherwise in the specific mix design comment.

⁎ The equivalent NaOH molarity is calculated as though the NaOH pellets were dissolved in the water and used in viscous solution form.

⁎⁎ Waste glass powder replaced steel slag.

**Table 3:** Mix design (percent by weight) and 7-day compressive strengths and densities of geopolymer mortars with viscous solutions of caustic soda-based (NaOH or NaOH-Na_2_SiO_3_) single or binary alkaline activators cured at 40°C temperature.

Mix Number	Aluminosilicate sources					Alkaline activators			Superplasticizer	Total*	Specific mix design Comment	Average density (kg m^−3^)	Average compressive strength (MPa)
	Steel slag	Waste solid brick powder	Waste glass powder	Waste concrete aggregate	Waste solid brick aggregate	Ca(OH)_2_ powder	12 M NaOH solution / Cementitious materials ratio	12 M NaOH-Na_2_SiO_3_ solution / Cementitious materials ratio					
39	32.1	3	–	40	17	7.5	0.62	–	0.4	100	There was collective grinding of the powdered materials. Specimens cured at 40°C for 7 days	2100	25
40	24.6	10	10	35	15	5	–	0.58	0.4	100	There was collective grinding of the powdered materials. Specimens cured at 40°C for 7 days	1927	17

⁎ Excludes the viscous alkaline activator solution/cementitious materials ratio.

## 4 Experimental Design, Materials and Methods

### 4.1 *Geopolymer component materials*

Waste concrete and waste red clay hollow and solid bricks were obtained from demolished structures in Doha, Qatar and used as aluminosilicate sources together with steel slag from the Qatar National Cement Company. The waste concrete and bricks were broken down and reduced to less than 4 mm particle size using appropriate sieve sizes. It is reported that the use of concrete waste powder has a detrimental impact on the fluidity and compressive strength of geopolymer mortars [10,11]. Hence, waste concrete was used as fine aggregate (75 µm to 4 mm), while the solid and hollow red clay bricks were used together with steel slag as aluminosilicate powders. Solid red clay brick (75 µm to 1.4 mm) was used as fine aggregate in some mixes (see Tables 1 – 3). The 1.4 mm maximum aggregate size was based on the capacity of a Gilson 2.25×3 inches laboratory jaw crusher used to break it down into granular form. Waste glass was also obtained from demolished structures, ground to powder form (< 75 µm) and used in two mixtures (Table 2, Mix 36 and Table 3, Mix 40). Small amounts of some other additives such as gypsum, microsilica and aluminum sulfate octadecahydrate [Al_2_(SO_4_)_3_.18H_2_O] were also added to some of the mixtures in Table 1, Table 2 for strength enhancement as these additives could enhance the geopolymerization or hydration process [12,13].

Different alkaline activators were considered. These include solid powder or ground pellet forms of Ca(OH)_2_, CaO, and Ca(OH)_2_-NaOH, NaOH-CaCO_3_ and Na_2_SiO_3_-Na_2_CO_3_-Ca(OH)_2_ mixtures, which entailed just adding water to the mix of aluminosilicate powders, additives and powdered alkaline activator (Tables 1 – 3). Two mixes included both solid powder Ca(OH)_2_ and viscous solutions of NaOH and NaOH-Na_2_SiO_3_ as alkaline activators (Table 3, Mixes 39 and 40). Since these mixes are the conventional two-part geopolymer, a viscous solution of NaOH is denoted as a single alkaline activator, while a viscous solution containing NaOH and Na_2_SiO_3_ is denoted as binary alkaline activator.

### 4.2 *Geopolymer mortar production and testing*

The powdered aluminosilicate sources, alkaline activators and additives were dry-mixed together with the waste concrete and brick aggregates, as applicable, and thoroughly mixed in a Hobart N50 5-Quart commercial stand mixer. Where applicable, a Hyperplast-ES910i (H-ES910i) superplasticizer was then added to water and mixed with the other geopolymer component materials for one-part geopolymer mortars that involved ‘just adding water’ to solid powdered alkaline activators. The addition of the superplasticizer was meant to reduce the water/cementitious material ratio and improve the mechanical performance, thereby keeping the mixtures at flowable water contents suitable for 3DCP. Consequently, the water-to-cementitious materials ratios were kept as low as possible for a given mortar mixture to make the mixture flowable and amenable for 3DCP similar to values optimized for actual 3D printing of geopolymer mortar from CDW in a related work [3]. The water/cementitious material ratio was determined as the ratio of the mass of mixing water added to the total mass of powdered materials - both aluminosilicate sources and alkaline activators.

A few conventional (two-part) geopolymer mixtures with viscous alkaline activator solutions (NaOH and NaOH-Na_2_SiO_3_) were considered. The NaOH-based solutions were prepared by dissolving NaOH pellets in tap water to reach predetermined molarity levels. The NaOH solution was then left to cool to room temperature before use due to the exothermic reaction (creates high heat levels) that occurs between NaOH and water. The liquid Na_2_SiO_3_ (based on 4 parts Na_2_SiO_3_ powder to 6 parts water) was then introduced into the NaOH-precursor slurry and mixed adequately. A similar procedure was followed in the work of Şahin et al. [14]. For consistency with the definition for the one-part (‘just add water’) geopolymers, the water/cementitious material ratio of the mixtures with viscous solution alkaline activator(s) was estimated as the ratio of the mass of water used in preparing the viscous alkaline solutions to the total mass of powdered/ground pellet materials, including the NaOH and Na_2_SiO_3_.

The geopolymer mortars formed were then placed into 50×50×50 mm molds in duplicates. After 24 hours, the specimens were demolded and cured at ambient temperature (20°C) or 40/50°C as applicable. The specimens were then tested for compressive strength after 7 days following ASTM C109 [15].

## Limitations

Since the data described were for preliminary range-finding experiments, which involved large numbers of mixtures, the mortars formed were tested in duplicates rather than triplicates to save on the use of materials, thus allowing for more materials to be used during further detailed investigations. It is for the same reason that other key performance parameters such as tensile strength, durability, and toughness, usually employed for evaluating the practical applicability of geopolymer mortars were not studied.

## Ethics statement

The authors have read and followed the ethical requirements for publication in Data in Brief and do confirm that the current work does not involve human subjects, animal experiments, or any data collected from social media platforms.

## CRediT authorship contribution statement

**Reginald B. Kogbara:** Conceptualization, Funding acquisition, Methodology, Investigation, Formal analysis, Writing – original draft. **Abdelrahman Al-Zubi:** Methodology, Investigation, Writing – review & editing. **Eyad A. Masad:** Conceptualization, Funding acquisition, Supervision, Writing – review & editing.

## Data Availability

Raw and processed data file for early-age strength of geopolymer mortars from waste concrete and bricks (Original data) (Figshare).
